# Complex karyotypes in hematologic disorders: a 12-year single-center study from Lebanon

**DOI:** 10.3389/fonc.2024.1480793

**Published:** 2024-10-24

**Authors:** Souraya Rammal, Farid Abou Abdallah, Charbel Attieh, Zeinab El Mounajjed, Warde Semaan, Alain Chebly

**Affiliations:** ^1^ Faculty of Medicine, Saint Joseph University of Beirut (USJ), Beirut, Lebanon; ^2^ Center Jacques Loiselet for Medical Genetics and Genomics (CGGM), Faculty of Medicine, Saint Joseph University of Beirut (USJ), Beirut, Lebanon

**Keywords:** hematologic disorders, cytogenetics, complex karyotype, chromosomal abnormalities, karyotype, Lebanon, cancer cytogenetics

## Abstract

Conventional cytogenetic analysis is an important tool for the diagnosis of many hematologic disorders (HD). A karyotype is designed as « complex » when several alterations are detected. However, there is no clear consensus on the exact definition of a complex karyotype (CK), and there is a lack of studies that exclusively analyze CK in the literature. Complex karyotypes were analyzed over a period of 12 years at the Jacques Loiselet Center for Medical Genetics and Genomics (CGGM) at Saint Joseph University in Beirut (USJ) in Lebanon. 255 CK were analyzed with their associated chromosomal abnormalities (CA) detected. Out of 255 patients, 59.22% were males with a mean age of 59 years. The most common anomaly associated with CK was hyperdiploidy with a prevalence of 22.41%, which is different from a previously published study. To our knowledge, this represents the largest series of CK, particularly within the Middle East region. This study underscores the critical role of conventional cytogenetics in detecting CK, ultimately contributing to improved management of HD. Further investigations focusing on CK are needed.

## Introduction

For numerous years, conventional cytogenetics has been utilized to understand the nature of hematologic disorders (HD) and the behavior of cancer cells. These conventional techniques continue to be extensively employed in clinical genetics laboratories to this day (1). Karyotyping is used to define clonal chromosomal abnormalities that often occur in hematologic neoplasms, including number anomalies such as monosomies and trisomies, and also structural anomalies such as deletions, inversions, translocations, and others. Identifying recurrent anomalies enables the definition of a specific prognosis and facilitates the selection of appropriate treatment options (1). Abnormal karyotypes can be simple showing a single abnormality or present many alterations, and thus are named complex karyotypes (CK).

Whether in the context of simple karyotypes or CK, recurrent cytogenetic anomalies are identified. For example, chronic myeloid leukemia (CML) mainly results from the translocation between the long arms of chromosomes 9 and 22 leading to the *BCR-ABL* fusion (Philadelphia chromosome) (2, 3). Tyrosine kinase inhibitors, such as imatinib, constitute a cornerstone of CML management; however, a poor response to treatment is observed in patients with CK (4). Furthermore, in patients with chronic lymphoid leukemia (CLL), several chromosomal alterations are reported, including a deletion on the short arm of chromosome 17 [(del (17p)] that can impact therapeutic choices (5). Additionally, among recurrent cytogenetic anomalies, translocation between the long arms of chromosomes 15 and 17 [t(15,17)] resulting in *PML-RARA* fusion is a characteristic of acute promyelocytic leukemia (APL, AML-M3) (6). Many other recurrent cytogenetic anomalies are reported in the setting of different HD.

The first studies on CK were performed by Berger et al. in the 1980s who described CK in acute non-lymphocytic leukemia as presenting three or more different chromosomal abnormalities (CA) in the malignant clone (7). Over the years, CK were analyzed in different HD. Indeed, the significance of karyotyping has been underscored by Yunis et al. who reported that the presence of three or more CA in myelodysplastic syndromes (MDS) was associated with a poor prognosis (8). Concerning acute myeloid leukemia (AML), Mrozek et al. defined ‘typical’ CK as CK showing the following abnormalities of 5q, 7q and/or 17p, however, their absence denotes an “atypical” CK (9). Furthermore, AML patients with typical CK were older, had more *TP53* mutations, and a shorter overall survival (OS) compared to AML patients with atypical CK.

Although there is no clear consensus on the exact definition of CK, the Francophone Group of Hematologic Cytogenetics (GFCH) described CK as abnormal karyotypes presenting three or more CA. Furthermore, GFCH defined subgroups according to the number of CA: low-CK with three CA, intermediate-CK with four, and highly-CK with five or more CA (10). The presence of CK at diagnosis holds a significant prognostic value in predicting response to treatment, relapse, and OS. However, interpreting CK remains challenging and varies among different hemopathies (10).

To our knowledge, there is a lack in studies that have exclusively investigated CK, focusing on their complexity, incidence, and associations. Based on our research, there is only one similar cytogenetic study that has been previously published. This study was carried out in Pakistan reporting data on 41 CK over a 6-years period (11). In the current study, we assessed the prevalence of CK in HD and compared their distribution among Lebanese patients. Additionally, we compared our results with those of the previously published study.

## Materials and methods

A total of 5681 hematologic cases were referred to the Jacques Loiselet Center for Medical Genetics and Genomics (CGGM), previously named the Medical Genetics Unit (UGM), at Saint Joseph University in Beirut (USJ), a tertiary referral center in Lebanon, between January 2011 and May 2023. Among these, 255 cases presented CK.

For all samples, we employed conventional cytogenetic procedures. Karyotyping was conducted on bone marrow aspirate or peripheral blood. Two cell cultures were established in RPMI 1640 medium supplemented with 10% FBS and 1% penicillin-streptomycin. Mitogens were introduced to the cell cultures as deemed necessary based on the diagnosis or suspicion, such as IL2+DSP30 for mature B-cell neoplasms, and PHAm for T-cell neoplasms. Cell cultures were incubated at 37°C with 5% CO_2_. After 24h, 48h or 72h, cells were harvested in accordance with standard cytogenetic methods, using KCl hypotonic treatment and ethanol-acetic acid 3V:1V fixative. Then, cytogenetic pellets were spread on Superfrost slides, followed by RHG banding. Fifteen to 25 R-banded metaphases were analyzed according to the recommendations of the “international system of human cytogenetic nomenclature” (ISCN 2016 or 2021) (12), using the IKAROS software (Metasystems, Germany). Additionally, we used GFCH cut-off values to classify CK according to the number of CA. *P* values less than 0.05 were considered statistically significant.

## Results

In our study, 255 CK were detected (4.48%). Among these, 151 (59.22%) were males and 104 (40.78%) were females, with a sex ratio of 1.5. The mean age was 59 years (median 65 years) ranging from 1 to 90 years; with 94.2% of adults.

**Figure 1:**
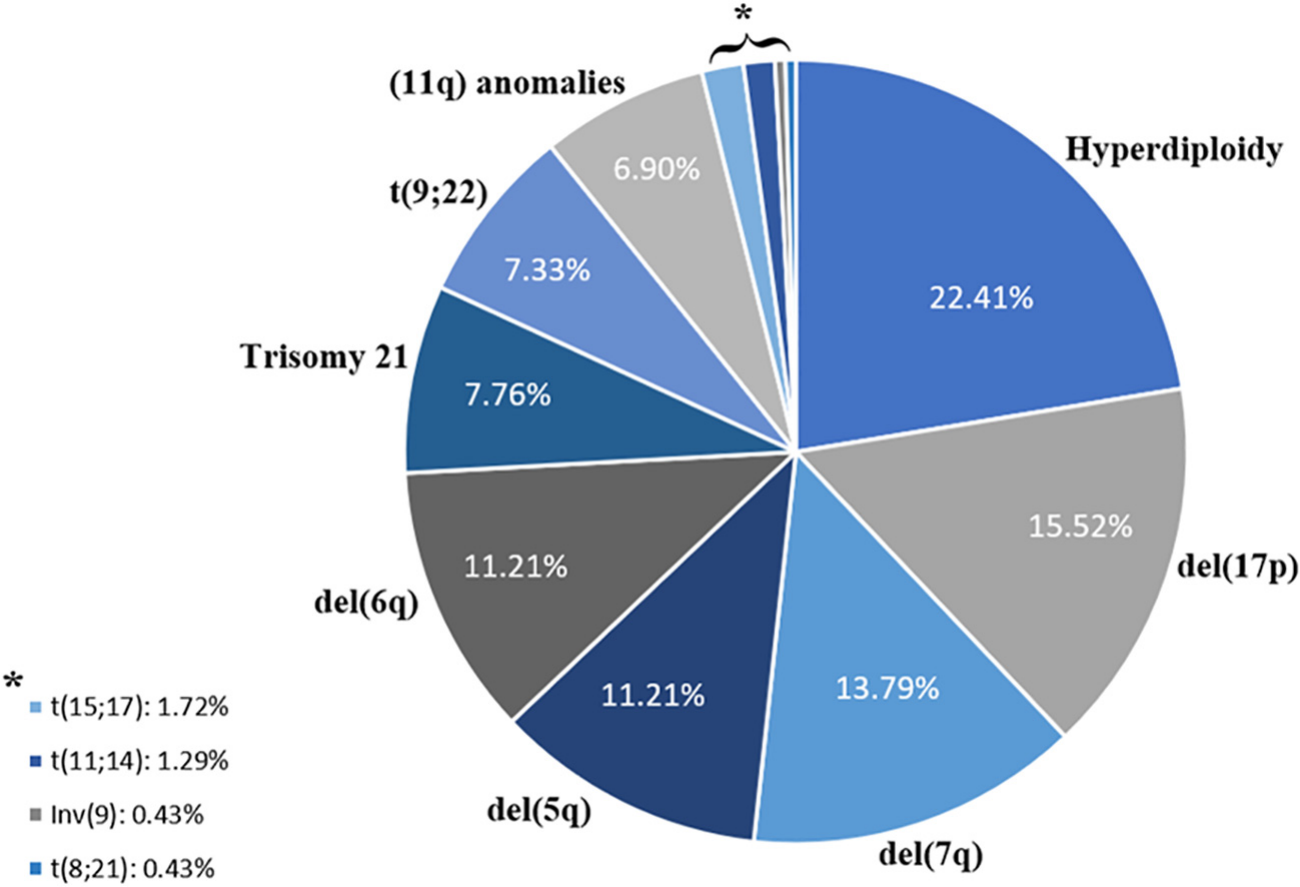
Distribution of chromosomal abnormalities associated with complex karyotypes.

Furthermore, we evaluated the recurrence of different CA detected in combination with CK. Our analyzes showed that hyperdiploidy was the most common CA detected, with a frequency of 22.41%, followed by 17p deletion (15.52%) and 7q deletion (13.79%) (**Figure 1**). **Table 1** illustrates the classification of the most prevalent anomalies detected among the various HD.

**Table 1:** Distribution of the frequent anomalies in CK among different HD.

Frequent anomalies in CK	Number of cases									
	CLL	MM	AML	MDS	Lymphomas	ALL	MPD	CML	Other HD	Total
Hyperdiploidy	7	15	5	3	5	7	2	0	13	57
del(17p)	12	2	6	3	7	1	0	1	7	39
del(7q)	5	2	9	2	8	5	0	0	4	35
del(5q)	0	0	8	13	0	3	1	0	3	28
del(6q)	3	4	4	5	8	1	1	0	2	28

ALL, acute lymphoblastic leukemia; AML, acute myeloid leukemia; CK, Complex Karyotype; CLL, chronic lymphocytic leukemia; CML, chronic myeloid leukemia; HD, Hematologic Disorders; MDS, myelodysplastic syndrome; MM, multiple myeloma; MPD, myeloproliferative disorders.

Interestingly, and regardless of the sex distribution, we observed that most of the CK in our study (68.24%) had five or more CA and are therefore classified as highly complex (**Table 2**). Furthermore, CLL presented the highest CA rates among all other HD in our study (14.90%), followed by multiple myeloma (MM) (12.55%) and AML (11.76%) (**Table 3**). On a side note, the ‘other HD’ group included patients referred for isolated leucopenia or anemia, monoclonal gammopathy of undetermined significance (MGUS), among other HD.

**Table 2:** Distribution of complex karyotypes according to the GFCH (Francophone Group of Hematologic Cytogenetics).

Number of Chromosomal Abnormalities	Classification of CK, according to the GFCH	Complex Karyotypes N (%)
3	Low-CK	41 (16.08)
4	Intermediate-CK	40 (15.69)
5	Highly-CK	174 (68.24)

**Table 3:** Distribution of complex karyotypes among hematologic diseases.

Hematologic Diseases	Number of CK	Percentage (%)
CLL	38	14.90
MM	32	12.55
AML	30	11.76
MDS	21	8.24
Lymphomas	21	8.24
ALL	17	6.67
MPD	15	5.88
CML	9	3.53
Other HD	72	28.24

ALL, acute lymphoblastic leukemia; AML, acute myeloid leukemia; CK, Complex Karyotype; CLL, chronic lymphocytic leukemia; CML, chronic myeloid leukemia; MDS, myelodysplastic syndrome; MM, multiple myeloma; MPD, myeloproliferative disorders.

On the other hand, our results did not show statistically significant differences in the distribution of CK between males and females, except for CML (p-value=0.01). In fact, a male predominance was observed in CML-CK (n=9), while zero females presented CK with CML in our study (**Supplementary Table 1**).

## Discussion

In the current study, we present, for the first time, comprehensive data from the Middle East concerning CK across a substantial series of hematologic neoplasms.

First, in comparison to the previously published study from Pakistan, the only similar study reporting on CK, we noticed that in our study we detected 4.48% of CK, slightly higher than the 3.4% reported by Waheed et al. (11). Furthermore, the male-to-female sex ratio in our study (1.5:1) differed significantly from the ratio of 4:1 reported in Pakistan. This divergence could be attributed to the small sample size in the Pakistani study (only 41 CK cases), and also to the different demographical characteristics among populations. Additionally, we noted an important difference in the mean age of CK patients: 59 years (median 65 years) in our study, versus 37 years (median 39 years) in the Pakistani study. This variation may be population-dependent and may be related to the increase in life expectancy in Lebanon, since life expectancy data in Lebanon align more closely with data collected from western populations such as the US (13).

Furthermore, while evaluating the association between recurrent CA and CK, our analyzes revealed that hyperdiploidy was the most common CA. This finding contrasts with the results reported by Waheed et al., where trisomy 21 was the most common CA followed by hyperdiploidy (11). This discrepancy is likely attributed to differences in demographic characteristics and the prevalence of various hematological disorders among populations. In this context, hyperdiploid karyotypes are significantly reported in several neoplasms, including (but not limited to) MM patients (14). Our study revealed an association between certain chromosome gains in cases of MM, notably identifying gains of chromosomes 3, 9, 11, 15, and 19 as the most frequently observed. Additionally, this CA is associated with a good response to treatment in children with Acute Lymphoblastic Leukemia (ALL) (15), but showed an unfavorable prognosis in cases of AML (10), depending on the associated cytogenetic abnormalities.

Regarding HD distribution, our findings diverge significantly from those reported in Pakistan, where the most prevalent diagnoses were MDS and AML (11). In our study, CLL emerged as the foremost diagnosis thus establishing itself as the most frequently encountered HD with CK.

In a brief review of the literature, we observed a decline in karyotyping requests for CLL cases over time, and that cytogenetic analyzes have predominantly shifted towards Fluorescence *In Situ* Hybridization (FISH) or microarray (16). The current study, in addition to our previous work on CLL, showed a low request for karyotyping in cases of CLL. Our data once again highlighted the importance of karyotyping in CLL, since CK regained its important prognostic value in CLL (17, 18), along with common abnormalities tested by molecular cytogenetics (deletion 17p, deletion 11q, deletion 13q and trisomy 12) (19, 20). In fact, Jondreville et al. (21) recommends a systematic karyotyping in patients with CLL before treatment because it can identify CK that are not detected by targeted techniques such as FISH. Additionally, several molecular biomarkers are important in CLL, such as the *IGHV* mutational status (22, 23).

The sample size of our study is important, but the main limitation of this study is the absence of survival and follow-up data from the patients. This information was not available for not all patients. However, based on the data that were accessible, most patients with CK in AML, ALL and MDS experienced poor outcomes and died due to the disease, adverse events or infections. In cases of CK-AML, Venetoclax was frequently used in combination therapies, yet a poor prognosis was still observed. T limitations of our study hindered our ability to draw conclusions regarding overall survival, treatment-free survival, or the worst prognosis linked to CK. However, our results still provide insightful cytogenomic information and can be considered as a basis for further investigations.

In conclusion, in the era of ‘molecular diagnostics’, it is clear that conventional cytogenetics still has room for the management and treatment of hematologic disorders. Despite significant advancements in the field, conventional karyotyping remains essential for detecting complex karyotypes. Emerging AI-driven technologies are poised to play a crucial role in the future (24). The current study is considered the first in the Middle East region and among a few published studies worldwide, with the largest sample size, highlighting the particularity of CK. To conclude, our study, which analyzes a representative sample size over a significant 12-year period, provides substantial credibility to our data. We believe these findings can serve as a valuable foundation for future clinical research focused on complex karyotypes, treatment strategies, and patient outcomes. 

## Data Availability

The original contributions presented in the study are included in the article/**Supplementary Material**. Further inquiries can be directed to the corresponding author.
