# Supplementary material for: Complex karyotypes in hematologic disorders: a 12-year single-center study from Lebanon
Source: Front Oncol. 2024 Oct 24;14:1480793. doi: 10.3389/fonc.2024.1480793 (PMC11540823; doi:10.3389/fonc.2024.1480793)
Supplement: Supplementary file 1 [file Table1.docx]

**Supplementary Table 1.** Distribution of the proportions of complex karyotypes between males and females.

| Hematologic Diseases |  | Sex N (%) | | p-value |
| --- | --- | --- | --- | --- |
|  | **Total** | **Male** | **Female** |  |
| CLL | 38 | 20 (52.63) | 18 (47.37) | 0.37 |
| MM | 32 | 24 (75.00) | 8 (25.00) | 0.05 |
| AML | 30 | 18 (60.00) | 12 (40.00) | 0.93 |
| MDS | 21 | 12 (57.14) | 9 (42.86) | 0.84 |
| Lymphomas | 21 | 13 (61.90) | 8 (38.10) | 0.79 |
| ALL | 17 | 7 (41.18) | 10 (58.82) | 0.12 |
| MPD | 15 | 7 (46.67) | 8 (53.33) | 0.31 |
| CML | 9 | 9 (100) | 0 (0) | **0.01** |
| Other HD | 72 | 41 (56.94) | 31 (43.06) | 0.64 |
| Total | 255 | 151 (59.22) | 104 (40.78) |  |

*ALL: acute lymphoblastic leukemia; AML: acute myeloid leukemia; CK: Complex Karyotype; CLL: chronic lymphocytic leukemia; CML: chronic myeloid leukemia; HD: Hematologic Disorders;* *MDS: myelodysplastic syndrome; MM: multiple myeloma; MPD: myeloproliferative disorders.*
